# Characterization of Φ2954, a newly isolated bacteriophage containing three dsRNA genomic segments

**DOI:** 10.1186/1471-2180-10-55

**Published:** 2010-02-19

**Authors:** Xueying Qiao, Yang Sun, Jian Qiao, Fabiana Di Sanzo, Leonard Mindich

**Affiliations:** 1 Department of Microbiology, The Public Health Research Institute Center, UMDNJ, Newark NJ, USA

## Abstract

**Background:**

Bacteriophage Φ12 is a member of the Cystoviridae and is distinct from Φ6, the first member of that family. We have recently isolated a number of related phages and five showed high similarity to Φ12 in the amino acid sequences of several proteins. Bacteriophage Φ2954 is a member of this group.

**Results:**

Φ2954 was isolated from radish leaves and was found to have a genome of three segments of double-stranded RNA (dsRNA), placing it in the Cystoviridae. The base sequences for many of the genes and for the segment termini were similar but not identical to those of bacteriophage Φ12. However, the host specificity was for the type IV pili of *Pseudomonas syringae *HB10Y rather than for the rough LPS to which Φ12 attaches. Reverse genetics techniques enabled the production of infectious phage from cDNA copies of the genome. Phage were constructed with one, two or three genomic segments. Phage were also produced with altered transcriptional regulation. Although the *pac *sequences of Φ2954 show no similarity to those of Φ12, segment M of Φ2954 could be acquired by Φ12 resulting in a change of host specificity.

**Conclusions:**

We have isolated a new member of the bacteriophage family Cystoviridae and find that although it shows similarity to other members of the family, it has unique properties that help to elucidate viral strategies for genomic packaging and gene expression.

## Background

Bacteriophage Φ6 was the first member of the Cystoviridae to be isolated [1]. In 1999 we isolated a number of phages that were members of the Cystoviridae [2]. Some were close relatives of Φ6 while others were rather distantly related in that they shared little or no base sequence similarity although their gene order was similar and they all contained genomes of three segments of dsRNA enclosed in a polyhedral shell that was, in turn, encased in a lipid-containing membrane. All members of this family have an inner core composed of 120 molecules of the major structural protein P1, 12 hexamers of the packaging NTPase P4, 12 molecules of polymerase P2 and about 30 molecules of auxilliary protein P7. The core is encased in a shell of protein P8 in all members except the Φ8 group. This is designated as the nucleocapsid. The nucleocapsid is covered by a lipid-containing membrane which has protein P9 as its major component and proteins P6 and P3 which determine host specificity. We proposed four groups represented by phages Φ6, Φ13, Φ12 and Φ8. The phages in the last three groups attached to host cells through rough LPS while the Φ6 group attached to type IV pili. We have recently isolated a new collection of phages and they seem to fit into the previously proposed groups with some important distinctions. In this paper we describe bacteriophage Φ2954 which has similarity to Φ12 [3] in the amino acid composition of several of its proteins but whereas Φ12 attaches to rough LPS, Φ2954 attaches to type IV pili. Φ2954 is of special interest in that it is dependent upon a host protein, glutaredoxin3 to activate its transcription upon initiating infection (unpublished results). It appears that different members of the Cystoviridae use different host proteins to activate or to regulate transcription [4]. The control of transcription in Φ2954 involves the nature of the first base of the segment L transcript while that of Φ6 and its close relatives involves the nature of the second base.

## Results and Discussion

**Figure 1:**
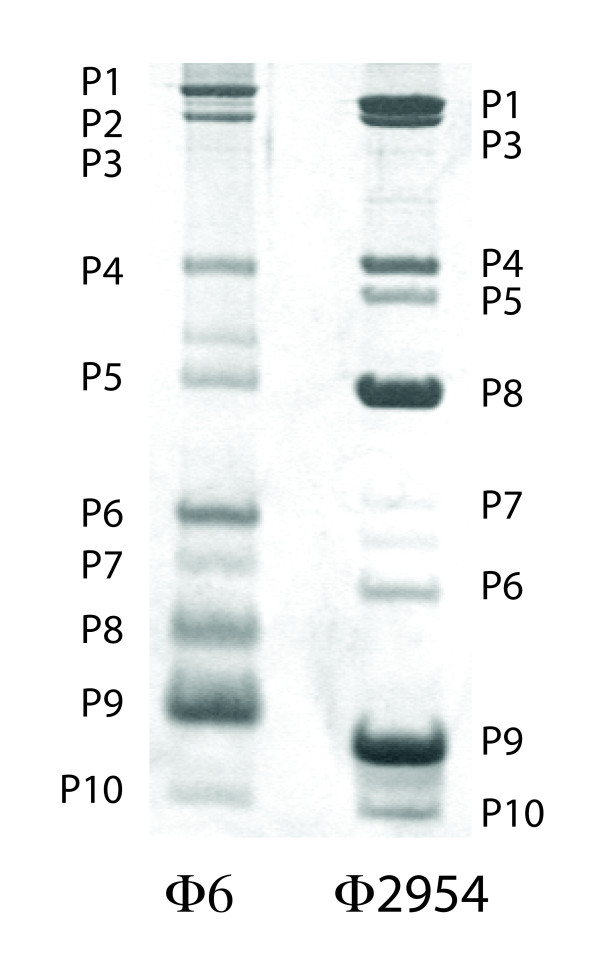
**Bacteriophage Φ2954 was purified by zone and equilibrium centrifugation in sucrose gradients and applied to an 18% polyacrylamide gel for electrophoresis**. The gel was stained with Coomasi blue. Purified Φ6 virions were displayed for comparison.

**Figure 2:**
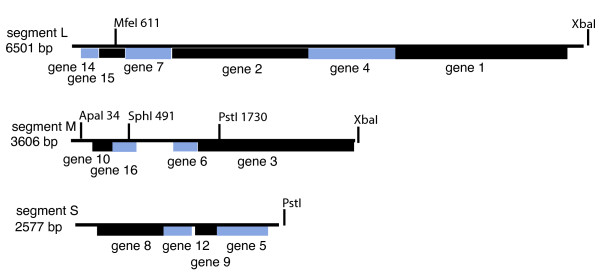
**Genetic maps of the genomic segments of Φ2954**. Restriction sites utilized in the construction of phage variants are shown. *PstI *and *XbaI *sites are present in the plasmid vectors for the cDNA copies.

**Figure 3:**
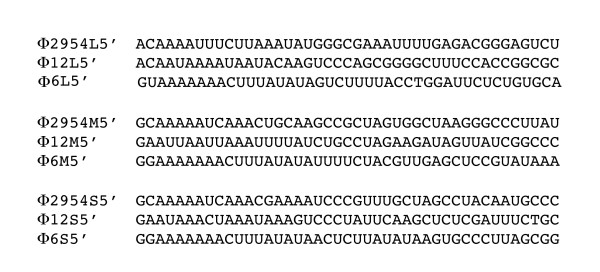
**Sequence comparisons at the 5' termini of transcripts of Φ2954, Φ12 and Φ6**. Note that in each case the sequence of L is different from those of S and M.

**Figure 4:**
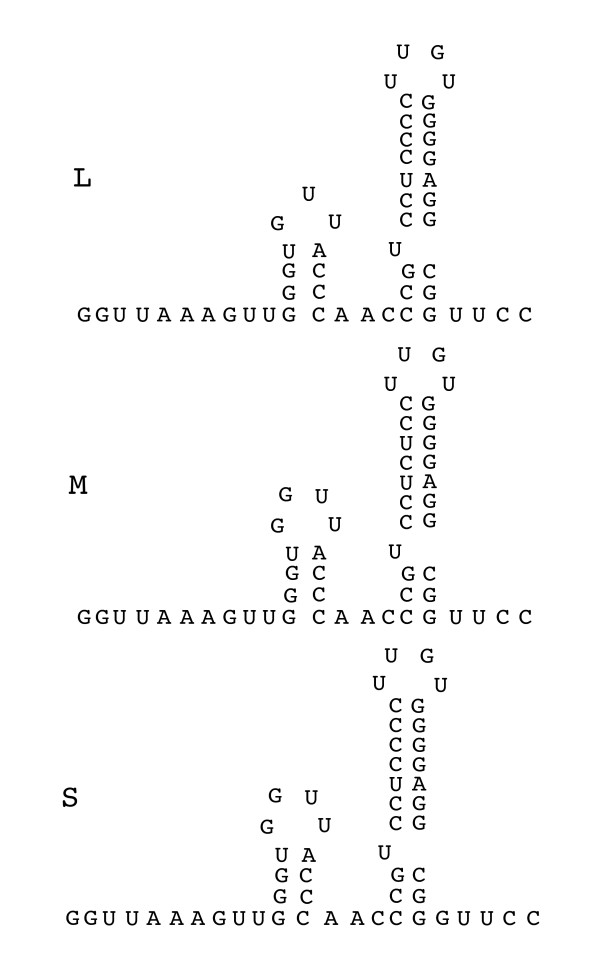
**Stem loop structures at the 3' termini of the Φ2954 transcripts**.

Twenty five new isolates of members of the Cystoviridae were obtained from the leaves of radish, carrot and onion plants. Five of the isolates showed similarity to previously isolated Φ12 [5] although their host ranges differed from that of Φ12. Radish leaves were incubated with LB broth. The liquid was mixed with a culture of *Pseudomonas syringae *LM2489 which is a rough LPS derivative of the original host strain for the cystoviruses [2]. Plaques were tested for sensitivity to chloroform. An isolate named Φ2954 was found to contain three segments of dsRNA. The sizes of the RNA segments differed from those of the known cystoviruses. The host range of the phage was similar to that of Φ6 in that it did not propagate on strains missing type IV pili but did propagate on strain HB10Y which has type IV pili and smooth LPS. Phage was purified by sedimentation and equilibrium banding in sucrose or Renocal (Bracco Diagnostics) gradients. Purified phage was analyzed by polyacrylamide gel electrophoresis (Fig. 1). The migration of the proteins was similar to that seen for most of the Cystoviridae and that of protein P8 was similar to that of Φ12 in that it appeared to have a molecular weight of 22 kd rather than that of 16 kd shown by most of the Cystoviridae. cDNA was prepared from the genomic dsRNA of the phage or from transcripts produced in vitro by nucleocapsids of the virus. cDNA was prepared using random hexamers or polyA tailing in conjunction with oligodT priming. The sequences were compiled into the maps shown in Figure 2. The sizes of the genomic segments were found to be 2578 bp, 3606 bp and 6501 bp respectively for segments S, M and L. Blast searches showed no significant nucleotide similarity with other phages but searches of amino acid sequence showed significant similarity to many of the gene products of bacteriophage Φ12 (Table 1) [6]. In particular, the amino acid sequence of the viral RNA polymerase, P2, was closely related to that of Φ12. Several of the differences shown by Φ12 relative to Φ6 were present in P2 of Φ2954. This was true of the regions in P2 of Φ6 at nucleotide positions K223 and R225; R268 and R 270; and S452 that deal with triphosphate binding and catalytic sites [7]. Moreover, the 5' terminal sequences of the segment transcripts resembled, but were not identical to those of the Φ12 genomic segments (Fig. 3). Φ12 differs from other members of the Cystoviridae in the base sequences at the 5' termini of plus strand copies of the genome. Whereas most examples have GG at the 5' termini of segments S and M and GU at the 5' end of L, Φ12 has GAA at the termini of segments S and M and ACAA at the 5' of segment L. Φ2954 has the sequence of GC at the 5' termini of segments S and M and ACAA at the 5' terminus of L. Bacteriophage Φ8 and its close relatives have identical sequences, GAAAUUU, at the 5' termini of all three transcripts [8]. The 3' sequences of the three plus strands contained a 55 base near identity at the terminus. This sequence produced a structure with two hairpin stem loops that differ in sequence from those of phi12 and other members of the Cystoviridae but probably function as protection against host exonucleases (Fig. 4) [9]. Amino acid similarity to some of the proteins of the Φ6 L segment was also found, but at a lower level than found for Φ12 (Table 1). An exception was the finding that protein P10 had striking similarity to P10 of Φ13, a phage that otherwise had little similarity to Φ2954 (Table 1). A strong relationship was found between the product of gene 5 and protein FlgJ (GI:71555478) of the host organism *P. syringae*. Protein P5 is a muramidase in all the Cystoviridae while FlgJ is a host flagellar protein that has peptidoglycan hydrolase activity. The similarity of Φ2954 P5 to FlgJ is greater than that of Φ2954 to that of P5 protein of any of the other cystoviruses, even Φ12. It seems clear that gene5 was derived from the host muramidase gene. The Cystoviridae are capable of acquisition of genetic material from the host. Although acquisition is much more likely if *pac *sequences are on the introduced RNA, we have shown acquisition in cases where *pac *sequences are not present [10].

**Table 1:** Comparison of amino acid sequences of Φ2954 proteins to those of Φ12, Φ6, Φ13 and FlgF^a^

Protein	Similarity to Φ12	Identity to Φ12	Similarity to Φ6	Identity to Φ6	Similarity to FlgF^b^
P1	60	40	nss		
P2	66	50	38	24	
P3	nss^c^		nss		
P4	63	45	41	25	
P5	47	25	38	24	54/36
P6	nss		nss		
P7	55	33	nss		
P8	45	29	nss		
P9	51	33	nss		
P10	nss		nss 71^d^	57^d^	
P16	nss	nss	nss		
P12	57	30	nss		
P14	nss		nss		
P15	nss				

a Needleman-Wunsch alignment

b *P. syringae *FlgJ glycosidase [GenBank AAZ34689.1]

c no significant similarity

d relationship to Φ13

The arrangement of the genes is similar to that of most of the Cystoviridae [11]. Polar relationships are evident for genes 8 and 12; and 9 and 5. The arrangement of genes 7 and 2 is consistant with a polar arrangement but gene 2 does seem to have a proper Shine-Dalgarno sequence [12]. In these cases the orfs for genes 12 and 5 do not have proper Shine-Dalgarno sequences associated with them (Table 2).

**Table 2:** Base sequences at orfs of Φ2954

**gene**	**sequence**
1	UAGGAAGUUUGAACCAUGGCUAGAAGAAUC
2	GCCGAGUGGCUCCGAAUGAAGGAUGACACU
3	UGCCAAGGGGUUAAUAUGUCAACCGCUCU
4	UCAAGGAAACCUUGUAUGAAGAUGUUACCG
5	GCCGGUUAAUCCGCGGUGAGCAAACAAGGC
6	CGACGACUCGGGAGUAUGCAACAGUAUCUG
7	GUAUGGGAGUGUAAAAUGGAUCUUAUUAAA
8	AACAAGGAGCAAGAAAUGGCUAAGCCACCC
9	UGGCAGGAGAUUCAUAUGUUCGCTAAAAGC
10	CGUAGUAGUGAAACCAUGAAUAAAGTTCTG
16	CUUCGGGUUGAGCACAUGGCCCAUGCCAGA
12	AACAUCGCCGCUCUGAUGGGUGCUGUAAAC
14	AGAGGUGUUUUCGAUAUGUUGAAAGUUCAG
15	CAUGAGGUCUUGCGAAUGAACACUUAUCAA

### Reverse genetics

**Figure 5:**
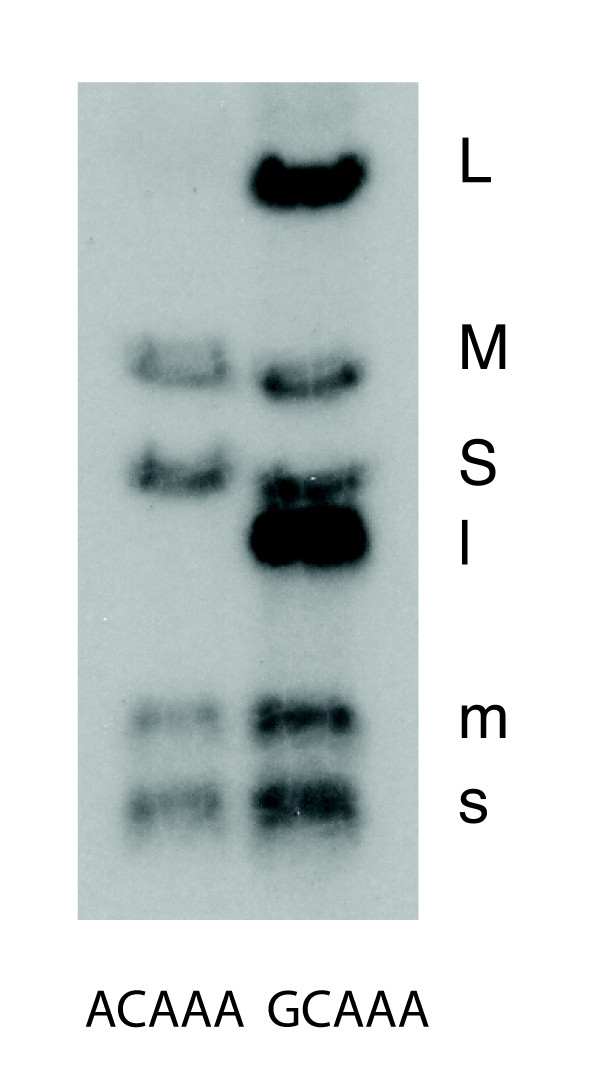
**In vitro transcription by nucleocapsids of Φ2954 having the normal 5' L sequence of ACAAA and a mutant, Φ3528, with the sequence GCAAA**.

The cDNA copies of the genomic segments were inserted into a derivative of plasmid pT7T319U (GenBank: U13870.1) that had the T7 RNA polymerase promoter replaced by the promoter of SP6 RNA polymerase so that transcription would start efficiently at the terminal G of segments S and M. The fidelity of the cDNA constructs was tested by their activity in the production of live virus resulting from their electroporation into strain LM3313. LM3313 is a derivative of LM2489 carrying plasmid pLM2790 that expresses the SP6 RNA polymerase. Three different constructs of the L segment were utilized; they contained 5' sequences beginning with the normal ACAA start, or with GACAA or GCAA. Segments M and S were normal (Figs. 2 and 3). The cDNA plasmids are ColE1 derivatives and unable to replicate in pseudomonads. The constructs with ACAA or GACAA produced about six thousand plaques from 10^10 ^cells while the construct of GCAA produced about one hundred plaques. The amount of transcript with ACAA would be expected to be much lower than that for GACAA, suggesting that its efficiency in plaque production would be greater than that for GACAA on a per molecule basis. While the phage resulting from the L segment with the normal 5' sequence of ACAA behaved identically to that of wild type Φ2954, the phage with the GCAA sequence showed novel transcription behavior. Whereas wild type Φ2954 nucleocapsids transcribe only genomic segments S and M in vitro; the mutant with GCAA instead of ACAA at the 5' terminus transcribes all three genomic segments in vitro (Fig. 5). The differences in template activity for genomic segment L as opposed to S and M is used by most members of the Cystoviridae to effect temporal regulation of transcription. In the case of Φ6 the L segment has the sequence GU at the 5' end of the plus strand while S and M have GG. The polymerase favors G over U as the second nucleotide and it is proposed that the second base is the first to be paired during transcription [13]. A host protein, YajQ, is able to alter this selection so as to enable active transcription of the L segment upon entry into the host cell [4]. A change of GU to GG in segment L of Φ6 results in in vitro transcription of segment L [14]. Although the structure of the polymerase of Φ2954 has not been studied, it seems likely that in this case the terminal nucleotide would be paired first and that G is preferred to A.

The host specificity of Φ2954 is different from that of its close relative Φ12; however it was possible to construct viable phage with a middle segment containing the *pac *sequence of Φ2954 and the genes 6 and 3 of Φ13. Gene 3 codes for the host attachment protein while gene 6 codes for its membrane bound anchor [15]. The plasmid pLM3575 has the 5' region of Φ2954M up to the *SphI *site at position 491 and the sequence of Φ13M from *SacII *at nucleotide 80. The resulting phage, Φ3010 does not plate on the normal host of Φ2954, HB10Y but does plate on strains that have rough LPS such as LM2509 or LM2489.

We have also constructed a plasmid with the *pac *sequence of Φ2954M and the genes 6 and 3 of Φ6. The resulting phage has the same plating properties as Φ2954 with respect to pilus attachment.

**Figure 6:**
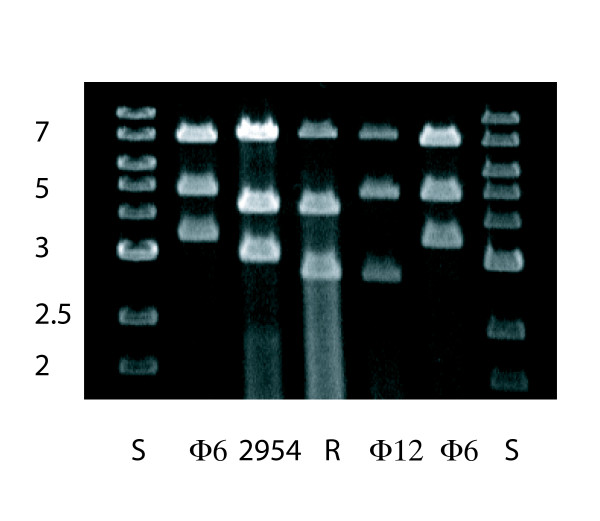
**Agarose gel electrophoresis of genomic segments of Φ12, Φ2954 and a Φ12 that has acquired segment M of Φ2954**.

Another test of the functionality of the cDNA copy of segment M was to determine whether bacteriophage Φ12 could acquire the transcript of this plasmid in order to change its host range. Plasmid pLM3497, which carries the cDNA copy of Φ2954 genomic segment M, was electroporated into strain LM3313 before infection with Φ12. These cells were plated along with those of HB10Y and plaques were obtained. These plaques plated on HB10Y but not on a strain missing the type IV pili. The genomic segments of these phages were consistent with the segments L and S of Φ12 and M of Φ2954 (Fig. 6). The finding that Φ12 is able to acquire segment M of Φ2954 is intriguing in that the *pac *sequences in M are very different for both phages. This is reminiscent of the case of bacteriophage Φ13 acquiring segment M of Φ6 in which case there is again very little sequence similarity in the *pac *sites [2]. This is so despite the observation that small changes in the *pac *sequences of Φ6 M or S drastically reduce the ability of Φ6 to acquire these segments [16].

The finding that it is possible to change the host attachment proteins is of special interest in that it shows that the proteins P6 and P3 are able to recognize viral membrane that contains the major membrane protein P9 of distantly related phages of the same family.

Another test of genomic packaging was the production of a genomic segment containing segments S and M joined together. The *ApaI *to *XbaI *segment of M was joined to the *PstI *site that is present in the vector following the 3' end of the cDNA copy of segment S. A plasmid pLM3662 containing this construct was able to donate its transcript to phage Φ3010 that contains segments S and L of Φ2954 and an M segment that has the *pac *sequence of Φ2954M but the genes of segment M of Φ13, a phage that plates on host cells containing rough LPS.

Electroporating plasmid pLM3695 into strain LM3313 produced a phage with the entire genome contained in a single segment. This plasmid contained the cDNA copies of the complete segment S with the sequence of segment M beginning with the *ApaI *site at position 34 to the *XbaI *site following its C terminus with segment L beginning with an *MfeI *site at position 611 that was converted to *XbaI*. The observation that phage were produced in high yield from this plasmid is consistent with the previous observations of the preparation of single segment genomes in Φ6 and Φ13. It also suggests that the open reading frames of genes 14 and 15, starting at 243 and 426, are not necessary for phage production.

## Conclusions

Φ2954 has a number of properties similar to other members of the Cystoviridae; however, it shows some interesting differences. In particular, it regulates transcription by altering the first nucleotide of the segment L transcript relative to those of segments S and M while most other cystoviruses regulate by altering the second nucleotide. The cDNA copies of the genome have been shown to be accurate and they allow manipulation of the structure of the genome. Φ2954 will be an important component in the investigation of the temporal control of transcription in the Cystoviridae.

## Methods

### Bacterial strains, phage and plasmids

LM2489 is a rough derivative of *P. syringae pv. phaseolicola *HB10Y (HB)[1] and was used as the primary host for plating Φ2954, Φ12 and Φ6.

Plasmid pLM1454 is a derivative of the cloning vector pT7T3 19U (GenBank: U13870.1). It was used for the cloning of cDNA copies of phage DNA produced by RTPCR.

### Media

The media used were LC and M8 {Sinclair, 1976 #80}. Ampicillin plates contained 200 mg of ampicillin per ml in LC agar.

### Enzymes and Chemicals

All restriction enzymes, T4 DNA ligase, T4 DNA polymerase, T4 polynucleotide kinase, Klenow enzyme, and Exonuclease BAL-31 were purchased from Promega, New England Biolabs and Boehringer Gmbh, Mannheim.

### Preparation of pure virions of Φ2954

Bacteriophage Φ2954 was harvested from soft LB agar plates. The soft agar was spun at 7000 rpm for 10 minutes at 4°C. 0.5 M NaCl and 10% PEG-6000 was added the supernatant liquid to precipitate the phage. The suspension was centrifuged; the pellet was resuspended in 0.5 ml of buffer B overnight at 4°C. Buffer B is composed of 10 mM KHPO_4_, 1 mM MgCl_2 _and 200 mM NaCl, pH 7.5.

The resuspended Φ2954 was then spun at 28,000 rpm for 70 minutes in a zone gradient of 10-30% Renocal in 200 mM Tris-HCl pH8, 200 mM NaCl, 1 mM MgCl2. The phage band was isolated and treated with PEG to precipitate the virions. The pellet was resuspended in 30 μl of the Tris buffer and extracted with phenol, ethanol precipitated and resuspended in 5 μl of DNA buffer.

### Preparation of cDNA. PolyA tailing

The RNA was denatured by boiling for 5 minutes and rapidly cooling with dry ice/ethanol. 5× polyA-polymerase buffer was added to the RNA along with ATP and yeast polyA polymerase (Amersham). The mixture was incubated at 30°C for 1 minute and transferred to ice and the reaction stopped with EDTA. The polyA-RNA was then extracted with phenol/chloroform and precipitated and resuspended in water.

### First strand synthesis

1 μl of phosphorylated oligo dT was added to 10 μl of polyA-RNA. After 5 minutes at 70°C the sample was cooled on ice for 5 minutes. Then 4 μl of 5× first strand buffer, 3 μl H_2_O, 40 u RNase inhibitor (RNasin) and 30 u AMV reverse transcriptase was added and incubated at 42°C for 1 hour. All products needed for the first and second strand synthesis were provided by the Promega cDNA kit (Universal Riboclone cDNA Synthesis System). The reaction products were stored at -70°C overnight.

### Second strand synthesis

After thawing the reverse transcribed RNA, 40 μl 2.5 × second strand buffer, 37.6 μl H_2_O, 0.8 u RNaseH and 23 u *E. coli *DNA polymerase I was added. After the second strand synthesis proceeded for 3 hours at 16°C, the *E. coli *DNA polymerase I was inactivated at 70°C for 10 minutes. Then T4 DNA polymerase was added for 10 minutes at 37°C to blunt the ends of the cDNA. The sample was then treated with phenol/chloroform, ethanol precipitated and resuspended in 2.5 μl H_2_O.

### Preparation of the vector used for cloning

pLM1454 was cut with *HincII*, dephosphorylated with shrimp alkaline phosphatase and then purified by electrophoresis, electroeluted, precipitated and resuspended in 20 μl TE buffer. The ligation mixture was composed of 2.5 μl Φ2954 cDNA, 0.5 μl vector, 0.5 μl 10 × ligation buffer, 0.5 μl 10 mM ATP and 2.5 u T4 DNA ligase. All products are provided by the Promega cDNA kit. Incubation was overnight at16°C. The ligation mixture was used to transform super competent Epicurean *E. coli *(Stratagene). The cells were resuspended in 100 μl SOC medium and plated out on LC plates with 40 μg/ml X-gal (5-bromo-4-chloro-3-indolyl-beta-D-galactopyranoside) and 200 μg/ml Ampicillin. White colonies were picked and small DNA preparations were made. The plasmids were cut with restriction enzyme *PvuII *and promising candidates were sequenced first with M13 primers and then with oligonucleotides prepared on the basis of the sequence found. At the point where it seemed that the ends of the segments were identified, we prepared cDNA copies by using RTPCR with oligonucleotides having sequences found in the first copies found. Sequencing was done at the New Jersey Medical School Sequencing Facility. The sequences of segments L, M and S were deposited in GenBank with respective accession numbers of [GenBank: FJ608823, FJ608824 and FJ608825].

### Preparation of complete cDNA plasmids

The cDNA pieces were assembled to form complete copies of the three genomic segments. In many cases, the connections could be made by using unique restriction sites made evident by the sequencing project. The ends of segments were prepared by using oligonucleotides with convenient restriction sites as primers for PCR reactions. Five plasmids were prepared, pLM3496, pLM3497, pLM3697, pLM3698 and pLM3691. They contain exact complete copies of genomic segments S and M in plasmid pT7T3 19U and three variants of segment L sequence. The sequences start at the first nucleotide of the SP6 RNA polymerase transcript.

### In vitro transcription with nucleocapsids

Nucleocapsids of Φ2954 were prepared from purified virions stripped of their lipid-containing membranes by treatment with two percent Triton X-100 [17]. Transcription was performed in magnesium buffers [18,19]. Labeling was with α-^32^P-UTP and products were analyzed by electrophoresis in agarose gels.

## Authors' contributions

JQ, XQ, YS and FD devised, carried out and analyzed the experiments described in this report. LM conceived the project and drafted the manuscript. All authors read and approved the final manuscript.
